# Secukinumab efficacy on resolution of enthesitis in psoriatic arthritis: pooled analysis of two phase 3 studies

**DOI:** 10.1186/s13075-019-2055-z

**Published:** 2019-12-04

**Authors:** Laura C. Coates, Johan K. Wallman, Dennis McGonagle, Georg A. Schett, Iain B. McInnes, Philip J. Mease, Lawrence Rasouliyan, Erhard Quebe-Fehling, Darren L. Asquith, Andreas E. R. Fasth, Luminita Pricop, Corine Gaillez

**Affiliations:** 1 Nuffield Department of Orthopaedics, Rheumatology and Musculoskeletal Sciences, Botnar Research Centre, University of Oxford, Windmill Road, Oxford, OX3 7LD UK; 2 Department of Clinical Sciences Lund, Rheumatology, Skåne University Hospital, Lund University, Lund, Sweden; 3 University of Leeds, Leeds, UK; 4 University of Erlangen-Nuremberg, Erlangen, Germany; 5 University of Glasgow, Glasgow, UK; 6 Swedish Medical Centre and University of Washington, Seattle, USA; 7 RTI Health Solutions, Barcelona, Spain; 8 Novartis Pharma AG, Basel, Switzerland; 9 Novartis Pharmaceuticals UK Ltd., Camberley, UK; 10 Novartis Sverige AB, Täby, Sweden; 11 Novartis Pharmaceuticals Corporation, East Hanover, USA

**Keywords:** Psoriatic arthritis, Enthesitis, Secukinumab, Interleukin 17A inhibitor, Anti-TNF, Biologics

## Abstract

**Background:**

Enthesitis is one of the psoriatic arthritis (PsA) domains. Patients with enthesitis are associated with worse outcomes than those without enthesitis. The effect of secukinumab on the resolution of enthesitis in patients with PsA was explored using pooled data from the FUTURE 2 and 3 studies.

**Method:**

Assessments of enthesitis through week 104 used the Leeds Enthesitis Index. These post hoc analyses included resolution of enthesitis count (EC = 0), median time to first resolution of enthesitis (Kaplan-Meϊer estimate), and shift analysis (as observed) of baseline EC (1, 2, or 3–6) to full resolution (FR), stable (similar or reduction of EC), or worse (EC > baseline). Efficacy outcomes (ACR, PASI, HAQ-DI, SF-36 PCS, and DAS28-CRP) were assessed in patients with or without baseline enthesitis. Results are reported for secukinumab 300 and 150 mg in the overall population and by prior TNFi treatment.

**Results:**

A total of 65% (466/712) of patients had baseline enthesitis. In the overall population, FR was achieved as early as week 16 in 65% (300 mg) and 56% (150 mg) versus 44% (placebo) patients, with further improvements to 91% (300 mg) and 88% (150 mg) at week 104. The majority (89%) of patients without enthesitis at baseline maintained this status at week 104. Median days to resolution of EC were shorter with secukinumab 300 and 150 mg versus placebo (57 and 85 vs 167 days, respectively). In patients with EC of 1 or 2, shift analysis from baseline to week 24 showed that more patients achieved FR with secukinumab 300 mg and 150 mg versus placebo, whereas no difference between secukinumab and placebo was shown in the more severe patients with EC of 3–6. Increases in proportions of patients with FR were observed with secukinumab irrespective of the severity of EC from baseline to week 104. Improvements in efficacy outcomes were similar in patients with or without enthesitis treated with secukinumab 300 mg.

**Conclusion:**

Secukinumab provided early and sustained resolution of enthesitis in patients with PsA over 2 years. Secukinumab 300 mg provided higher resolution than 150 mg in patients with more severe baseline EC and showed similar overall efficacy in patients with or without enthesitis.

**Trial registration:**

FUTURE 2: ClinicalTrials.gov, NCT01752634 (date of study registration: December 19, 2012), and EudraCT, 2012-004439-22 (date of study registration: December 12, 2012)

FUTURE 3: ClinicalTrials.gov, NCT01989468 (date of study registration: November 21, 2013), and EudraCT, 2013-004002-25 (date of study registration: December 17, 2013)

## Background

Psoriatic arthritis (PsA) is a chronic inflammatory disorder, associated with musculoskeletal manifestations (peripheral arthritis, spondylitis, enthesitis, and dactylitis) and extra-musculoskeletal manifestations (skin and nail disease) [1, 2], which has significant impact on health-related quality of life (HRQoL) and disability [3]. Of these, enthesitis is a clinical, periarticular manifestation seen both at early and late phases of the disease [4–6] which can differentiate PsA from rheumatoid arthritis [2, 7]. Enthesitis is defined as inflammation of tendon, ligament, or joint capsule insertion sites to bone and may be important in PsA development [2, 8]. Entheses are anatomically, functionally, and physiologically associated with synovia and form the “synovio-entheseal complex” which are comprised of soft and hard tissue. The enthesis organ dissipates stress, which may be a potential triggering mechanism of enthesitis and, ultimately, PsA [5, 9, 10]. About 30–50% of patients with PsA suffer from enthesitis based on standard clinical examination, which may, however, underestimate or misinterpret such symptoms [8], since imaging studies have revealed the prevalence rate to be as high as 70% [11].

Type 3 innate lymphoid cells and γ훿T cells are present at entheseal sites which on activation stimulate the production of interleukin (IL)-17 [4, 12]. The IL-17 pathway augments the influx and activation of neutrophils which are the effector cells of entheseal inflammation, and stimulates the release of proteases and reactive oxygen species that lead to pain response during entheseal inflammation [4, 8].

Enthesitis is frequently associated with increased pain, fatigue, physical disability, structural damage, and reduced work productivity compared to patients without enthesitis [13, 14]. According to the Group for Research and Assessment of Psoriasis and Psoriatic Arthritis (GRAPPA), enthesitis reflects higher disease burden and represents one of the six clinical core domains requiring diagnosis, assessment, and treatment in PsA [1, 15]. The recently updated American College of Rheumatology/National Psoriasis Foundation guideline has recommended treatment with IL-17 or IL-12/23 inhibitor in PsA patients with predominant enthesitis who have severe psoriasis or contraindications to first-line treatment with tumor necrosis factor inhibitors (TNFi) [16].

Secukinumab, a fully human, monoclonal IgG1κ antibody that directly inhibits IL-17A, has been shown to provide significant and sustained improvement in the different facets of active PsA [17–20]. In the FUTURE 2 and FUTURE 3 studies, secukinumab demonstrated early and sustained clinical efficacy in PsA patients up to 4 years [18–21]. Secukinumab 300 and 150 mg provided rapid and sustained resolution of enthesitis in ~ 70% of patients with enthesitis at baseline through 4 years in FUTURE 2 [18, 19, 21] and in ~ 50% patients through 1 year in FUTURE 3 [20].

Herein, we present a comprehensive post hoc analysis using pooled data from the FUTURE 2 and FUTURE 3 studies over 2 years, to further evaluate the effect of secukinumab on (a) enthesitis count (EC; defined by the Leeds Enthesitis Index [LEI]), (b) time to resolution of enthesitis, (c) shift in baseline EC (1, 2, or 3–6) to resolution, and (d) the occurrence of enthesitis in patients with no enthesitis at baseline. We also evaluated if clinical outcomes were similarly improved with secukinumab irrespective of the presence of enthesitis at baseline.

## Methods

### Study design and patients

FUTURE 2 (NCT01752634) is a 5-year and FUTURE 3 (NCT01989468) a 3-year, randomized, double blind, placebo-controlled phase 3 study [18, 20]. Details of the study designs and inclusion and exclusion criteria are reported elsewhere [18, 20]. Briefly, patients aged ≥ 18 years who met the ClASsification criteria for Psoriatic ARthritis (CASPAR) and had active disease (defined as at least three tender joints and at least three swollen joints) with inadequate response/intolerance to non-steroidal anti-inflammatory drugs (NSAIDs), disease-modifying anti-rheumatic drugs (DMARDs), or up to three TNFi agents were included. Key exclusion criteria were as follows: prior use of any biologics other than TNFi, active inflammatory diseases other than PsA, active infection in the 2 weeks before randomization, evidence of tuberculosis, and history of malignant disease within the past 5 years.

FUTURE 2 comprised 397 patients with active PsA who were randomized (1:1:1:1) to receive s.c. secukinumab 300, 150, or 75 mg or placebo at baseline; weeks 1, 2, 3, and 4; and once every 4 weeks thereafter. Placebo-treated patients were re-randomized to receive s.c. secukinumab 300 or 150 mg either at week 16 (non-responders with < 20% improvement from baseline in tender and swollen joint counts) or week 24 (responders with ≥ 20% improvement). In FUTURE 3, 414 patients with active PsA were randomized (1:1:1) to receive self-administered s.c. secukinumab 300, 150 mg, or placebo at baseline; weeks 1, 2, 3, and 4; and every 4 weeks thereafter. Placebo-treated patients were re-randomized to s.c. secukinumab 300 or 150 mg either at week 16 (non-responders) or 24 (responders). In this post hoc analysis, data were pooled from the FUTURE 2 and FUTURE 3 studies based on the presence or absence of clinical enthesitis (as defined by the LEI) at baseline.

The studies were conducted in compliance with the Declaration of Helsinki [22], International Council for Harmonization Guidelines for Good Clinical Practice, and local country regulations. The studies were approved by institutional review boards or independent ethics committees at each participating center. Written informed consent was obtained from all enrolled patients. Data were collected in accordance with Good Clinical Practice guidelines by the study investigators and were analyzed by the sponsor. The baseline demographics and clinical characteristics were compared in patients with and without enthesitis at baseline.

### Assessments

#### Leeds Enthesitis Index

In this post hoc analysis, patients were grouped based on the presence or absence of enthesitis at baseline as defined by the LEI, a validated instrument that uses six sites for evaluation of enthesitis (left and right sides): lateral epicondyles of the humerus, Achilles tendon insertions, and medial femoral condyles. The LEI is a reliable index showing good correlation with other enthesitis indices of PsA and can distinguish between patients with and without active enthesitis [8, 23, 24]. Tenderness on examination was recorded as either present (1) or absent (0) at each of the six sites, for an overall score range of 0–6 with higher scores indicating greater enthesitis burden. If enthesitis was present at any of the six sites at baseline, the patient was counted as a patient with enthesitis [23].

#### Resolution and time to first resolution of enthesitis

Among patients with enthesitis at baseline, the Kaplan-Meϊer analyses were employed (1) to calculate the proportion of patients with resolution of enthesitis at weeks 16, 52, and 104 and (2) to estimate the median time to first resolution of enthesitis (i.e., first assessment time with EC = 0).

#### Shift analysis of EC from baseline to weeks 24, 52, and 104

Shift analysis was performed to analyze the resolution of enthesitis at weeks 24, 52, and 104 based on severity of enthesitis categories at baseline, i.e., mild, EC = 1; moderate, EC = 2; or severe, EC = 3–6. There were three mutually exclusive resolution criteria in this analysis: full resolution (EC = 0), stable including those with EC partly improved (0 < EC ≤ baseline EC), and worse (EC > baseline EC).

#### Heat map analysis

Individual patient status of resolution of EC through week 104 by treatment arms was visualized using heat map analysis. All patients with enthesitis at baseline were followed until the end of study or discontinuation. Red shading was used for EC ≥ baseline, yellow shading for partial resolution with an EC < baseline, and green shading for full resolution with an EC = 0.

#### New enthesitis sites in patients without enthesitis at baseline

In patients without enthesitis at baseline, the number of sites developing new enthesitis (ES) was assessed through week 104.

#### Relationship between baseline enthesitis status and outcomes across multiple clinical domains

The relationship between the presence or absence of enthesitis and different efficacy outcomes was analyzed in patients with or without enthesitis at baseline after adjusting for confounding baseline characteristics at weeks 16 and 104. The efficacy outcomes included the following: proportion of patients achieving American College of Rheumatology (ACR) 20, 50, and 70 response rates; Psoriasis Area and Severity Index (PASI) 75 and 90 response rates; and mean change from baseline in Health Assessment Questionnaire Disability Index (HAQ-DI), Short Form 36 Physical Component Summary score (SF-36 PCS), and 28-Joint Disease Activity Score using C-reactive protein (DAS28-CRP). Unadjusted analyses were also performed in patients with or without enthesitis.

#### Statistical analysis

Data are presented for secukinumab 300 and 150 mg (approved doses) up to week 104, and for placebo only up to week 16/24 (i.e., at weeks 52 and 104, data were analyzed only for patients originally randomized to secukinumab). The analysis on resolution and time to first resolution of enthesitis was done in the overall population, as well as split into TNFi-naïve and TNFi-IR (patients with an inadequate response or intolerance with prior use of up to three TNFi) subgroups. The Kaplan-Meϊer estimate accounted for censoring, dropouts, and loss to follow-up. Patients randomized to the secukinumab arms were followed until their last visit or loss to follow-up while patients in the placebo arm were censored at 168 days (24 weeks) of follow-up. The *Y*-axis on the Kaplan-Meϊer figures represent the survival function, which was defined as the proportion (%) of patients who had not yet experienced resolution at the particular time on the *X*-axis, after accounting for dropouts and censoring. The survival function was calculated using the product-limit formula, which was the proportion (%) of patients who had not yet experienced resolution at a particular time multiplied by the percentage at all previous times when enthesitis occurred. The percentage of patients with resolution at weeks 16, 52, and 104 were computed as 1 minus the survival function estimates at days 112, 365, and 729, respectively. The shift analysis on EC was performed from baseline to weeks 24, 52, and 104 according to EC severity at baseline (1, 2, 3–6) using as observed analysis in patients with data available at both visits.

Heat map analysis used the last observation carried forward (LOCF) to impute status between scheduled visits with available data at baseline and weeks 8, 16, 24, 52, and 104.

Clinical and patient-reported outcomes were evaluated by enthesitis status at baseline and at weeks 16 (all treatment groups) and 104 (secukinumab groups only) using as observed data. To adjust for potential confounding variables, logistic regression and analysis of covariance (ANCOVA) were employed to analyze binary and continuous variables, respectively. In each model, independent variables included treatment group, enthesitis status at baseline, gender, DAS28-CRP at baseline, HAQ-DI at baseline, prior TNFi status, and the interaction between treatment group and enthesitis status at baseline.

## Results

### Demographic and baseline disease characteristics

A total of 712 patients were included in this pooled analysis, of which 466 (65%) patients were diagnosed with enthesitis at baseline (secukinumab 300 mg, 144/239; 150 mg, 159/238; and placebo, 163/235). Demographics were generally comparable across groups except for a slightly higher proportion of females among the patients with baseline enthesitis. The mean time since first diagnosis of PsA in the pooled analysis was > 6 years in both the enthesitis and without enthesitis groups. Around two thirds of patients were naïve to TNFi therapy. On average, patients with enthesitis displayed three tender entheses at baseline and the mean EC was comparable between the secukinumab (3.0 and 3.2 with 300 and 150 mg, respectively) and placebo groups (3.0). The enthesitis group had more active disease at baseline, as reflected by numerically higher tender and swollen joint counts, patient and physician global assessment scores, DAS28-CRP, and HAQ-DI scores than patients without enthesitis (Table 1).

**Table 1:** Demographics and baseline characteristics of patients with and without enthesitis at baseline

Characteristic	With enthesitis at baseline			Without enthesitis at baseline		
	Secukinumab		Placebo (*N* = 163)	Secukinumab		Placebo (*N* = 72)
	300 mg (*N* = 144)	150 mg (*N* = 159)		300 mg (*N* = 95)	150 mg (*N* = 79)	
Age (years), mean (SD)	48.3 (12.5)	48.0 (11.8)	48.9 (13.3)	46.5 (13.7)	48.7 (12.8)	50.4 (11.9)
Female, %	55	53	69	44	48	33
Weight (kg), mean (SD)	88.1 (21.0)	90.8 (19.4)	82.9 (18.9)	83.8 (15.1)	84.1 (17.8)	85.3 (16.4)
Caucasian, %	95	93	97	96	92	96
TNFi-naïve, %	68	63	64	67	72	72
Time since first diagnosis of PsA (years), mean (SD)	7.7 (8.7)	7.4 (8.8)	6.8 (7.0)	8.2 (8.4)	6.7 (7.6)	7.1 (8.0)
Number of enthesitis sites, mean (SD)	3.0 (1.7)	3.2 (1.7)	3.0 (1.6)	0	0	0
Tender joint total score (78 joints), mean (SD)*	23.5 (15.6)	27.0 (19.8)	26.3 (18.4)	14.6 (9.6)	16.9 (13.6)	14.1 (11.2)
Swollen joint total score (76 joints), mean (SD)*	10.3 (6.9)	12.2 (10.1)	12.1 (10.3)	9.3 (7.4)	10.0 (8.1)	8.6 (7.1)
DAS28-CRP, mean (SD)	4.8 (1.0)	4.9 (1.1)	4.9 (1.1)	4.3 (1.0)	4.4 (1.1)	4.3 (0.9)
HAQ-DI, mean (SD)	1.3 (0.7)	1.2 (0.6)	1.3 (0.6)	1.0 (0.6)	1.2 (0.6)	1.1 (0.7)
Patient global assessment, mean (SD)	*N* = 142, 62.6 (18.7)	*N* = 156, 61.6 (21.6)	*N* = 161, 62.2 (20.1)	*N* = 95, 56.6 (21.5)	*N* = 79, 59.1 (19.9)	*N* = 72, 53.1 (19.9)
Physician global assessment, mean (SD)	*N* = 143, 54.4 (18.3)	*N* = 158, 57.0 (16.4)	*N* = 163, 57.1 (15.7)	*N* = 94, 51.0 (16.8)	*N* = 79, 53.6 (17.0)	*N* = 72, 49.9 (19.4)

*DAS28-CRP* 28-Joint Disease Activity Score count using C-reactive protein, *HAQ-DI* Health Assessment Questionnaire Disability Index, *PsA* psoriatic arthritis, *SD* standard deviation, *TNF* tumor necrosis factor

*In case of joints for which the data was not available, the observed count of the joints was scaled up proportionately

### Resolution of EC in patients with enthesitis at baseline

**Fig. 1:**
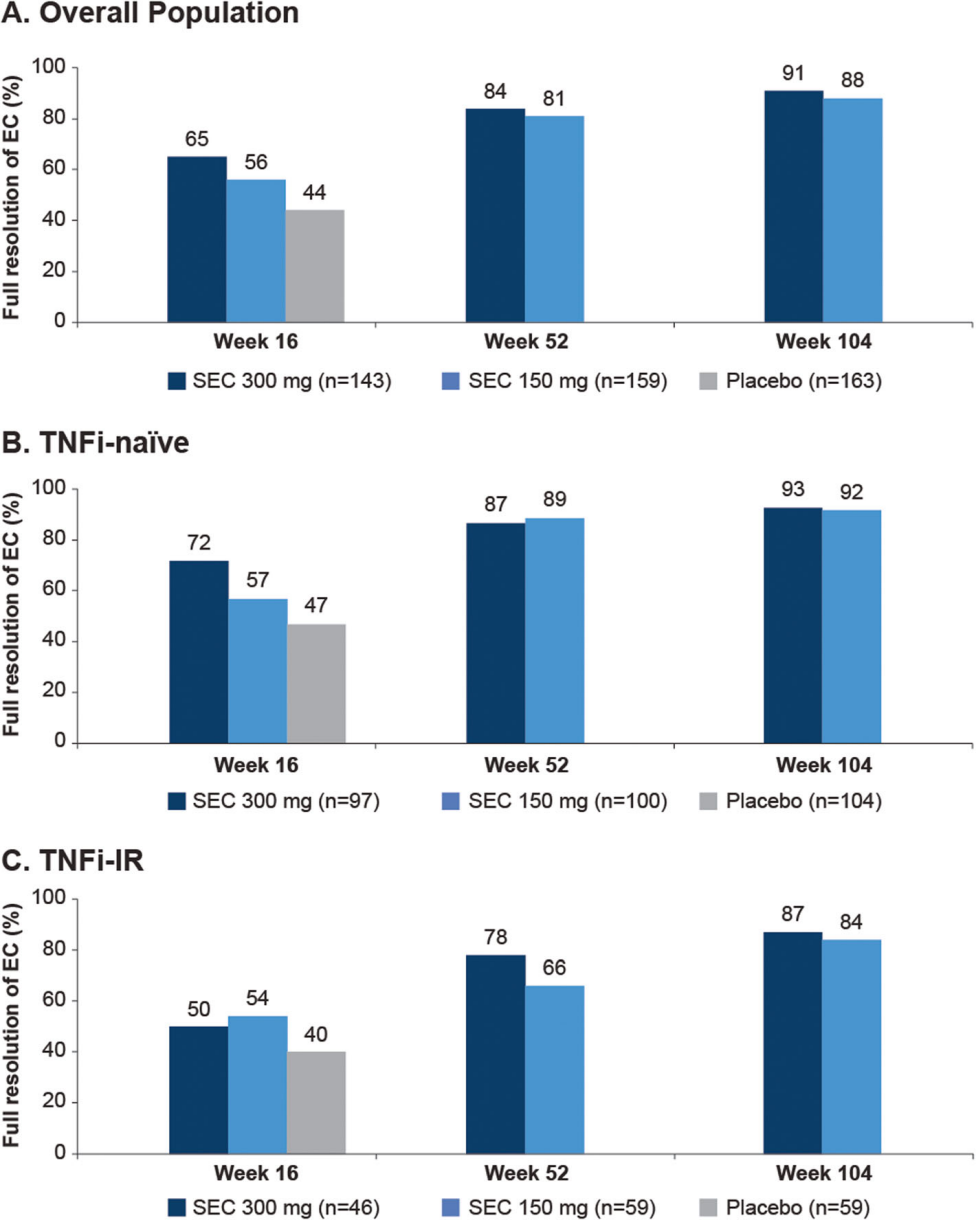
Proportion of patients with enthesitis at baseline achieving full resolution over 104 weeks. Data shown for overall population (**A**), TNFi-naïve (**B**), and TNFi-IR (**C**) subpopulations. *n*, number of patients with enthesitis at baseline. Proportion of patients with resolution at weeks 16, 52, and 104 were based on the survival analysis (Kaplan-Meϊer estimates) at days 112, 365, and 729, respectively. Placebo patients were censored at day 168 of follow-up. EC, enthesitis count; FR, full resolution; IR, inadequate responder; SEC, secukinumab; TNFi, tumor necrosis factor inhibitor

The Kaplan-Meϊer analysis showed that 65%, 56%, and 44% of patients in the overall population treated with secukinumab 300, 150 mg, and placebo, respectively, achieved full resolution of EC at week 16. This further improved to 91% and 88% with secukinumab 300 mg and 150 mg, respectively, at week 104 (Fig. 1). The magnitude of response was higher with secukinumab 300 mg than 150 mg. A high proportion of secukinumab treated patients achieved resolution of EC in both TNFi-naïve (300 mg and 150 mg, 72% and 57% vs 47% placebo [week 16]; 93% and 92% week 104]) and TNFi-IR patients (300 mg and 150 mg, 50% and 54% vs 40% placebo [week 16]; 87% and 84% [week 104]), with numerically higher responses in TNFi-naïve than TNFi-IR patients (Fig. 1).

### Time to first resolution of enthesitis

**Fig. 2:**
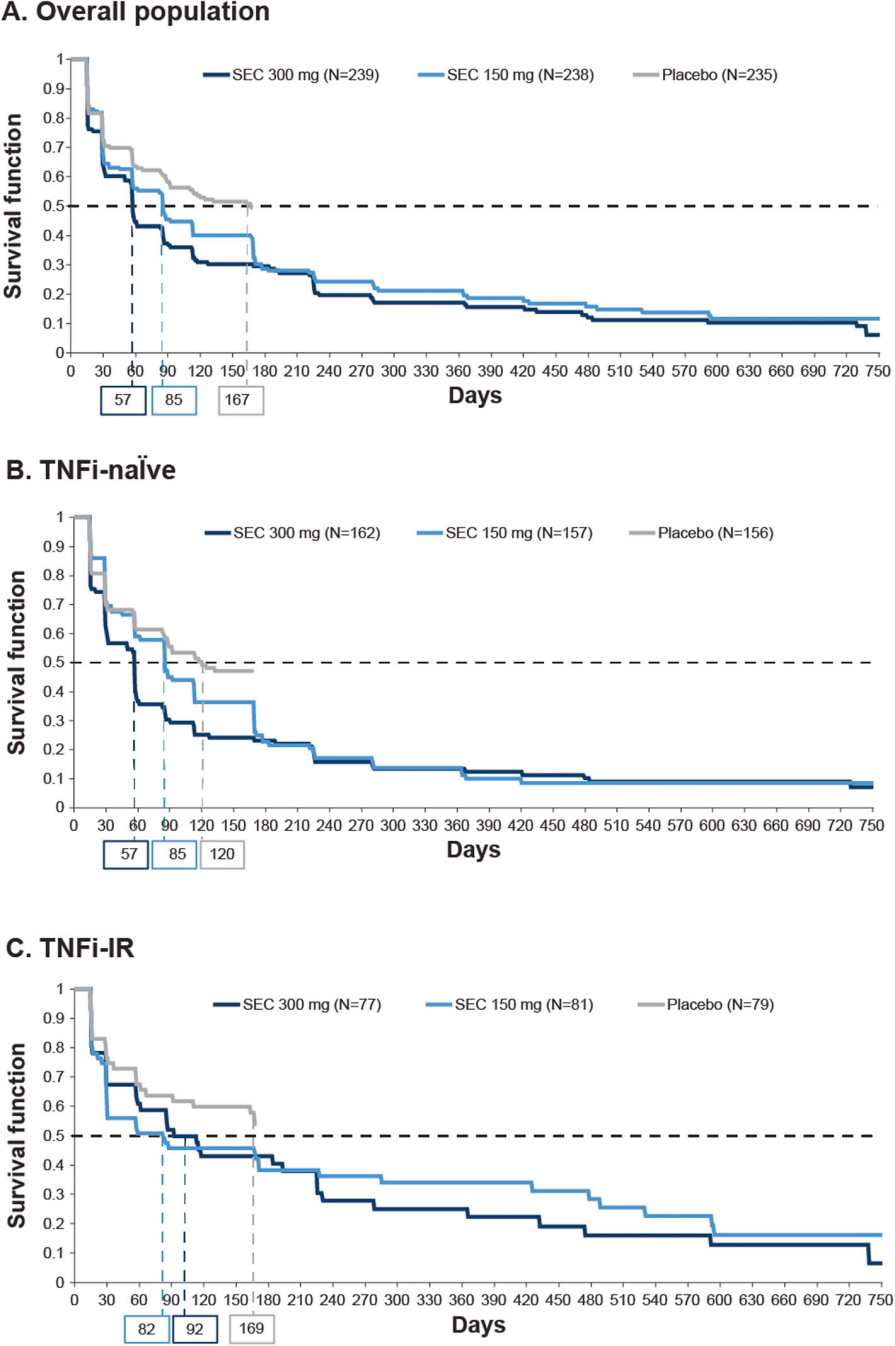
Time to resolution of enthesitis in patients with enthesitis at baseline. Data shown for overall population (**A**), TNFi-naïve (**B**), and TNFi-IR (**C**) subpopulations. Percentages of patients with resolution at weeks 16, 52, and 104 were derived as 1 minus the survival function at days 112, 365, and 729, respectively (Kaplan-Meϊer plot). Placebo patients were censored at day 168 of follow-up. IR, inadequate responder; SEC, secukinumab; TNFi, tumor necrosis factor inhibitor

The Kaplan-Meϊer plots of time to first resolution of enthesitis were presented for the overall population and split into TNFi-naïve and TNFi-IR subgroups (Fig. 2). The median days to resolution of EC in patients with enthesitis at baseline were shorter with secukinumab than placebo in the overall population (secukinumab 300 and 150 mg vs placebo, 57 and 85 vs 167 days), TNFi-naïve patients (57 and 85 vs 120 days), and TNFi-IR patients (92 and 82 vs 169 days) (Fig. 2), with resolution being faster in patients treated with secukinumab 300 mg versus 150 mg in the overall population and TNFi-naïve patients.

### Shift analysis in EC from baseline to weeks 24, 52, and 104

**Fig. 3:**
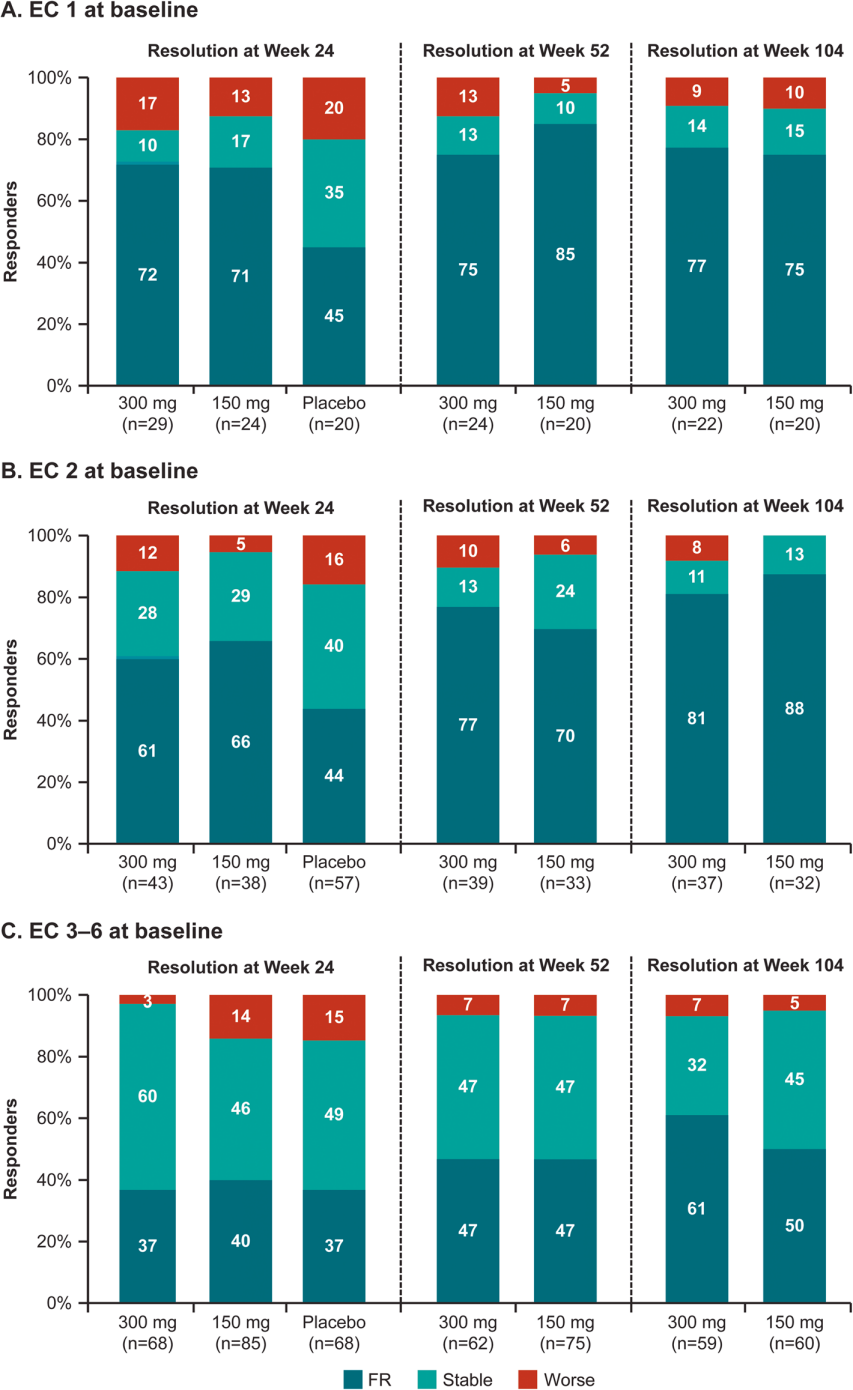
Shift analysis of enthesitis count from baseline to weeks 24, 52, and 104. **A**, **B**, and **C** represents EC = 1, 2, and 3–6 at baseline, respectively. EC 3–6 group includes patients with baseline EC 3, 4, 5, or 6. Shift analysis on resolution of EC from baseline to week 24/52/104 is categorized based on the three criteria of resolution: FR (EC = 0), stable (0 < EC ≤ baseline EC), and worse (> baseline EC). EC, enthesitis count; FR, full resolution; *n*, number of patients who completed week 24 and had EC available at both baseline and week 24

In patients with EC = 1 at baseline, 72% (secukinumab 300 mg), 71% (secukinumab 150 mg), and 45% (placebo), respectively, achieved full resolution at week 24, which increased to 77% (secukinumab 300 mg) and 75% (secukinumab 150 mg) at week 104 (Fig. 3). Similarly, in patients with EC = 2 at baseline, the response rates for full resolution at week 24 (61% [300 mg], 66% [150 mg] vs 44% [placebo]) further improved at week 104 (81% and 88% with secukinumab 300 mg and 150 mg, respectively). In contrast, in patients with severe enthesitis at baseline (EC = 3–6), no difference was seen in terms of full resolution between secukinumab 300 mg, 150 mg, and placebo (37%, 40% vs 37%) at week 24. By weeks 52 and 104, 47% and 61% (secukinumab 300 mg) and 47% and 50% (secukinumab 150 mg) of patients with EC = 3–6 at baseline had attained full resolution, respectively (Fig. 3).

### Heat map analysis

**Fig. 4:**
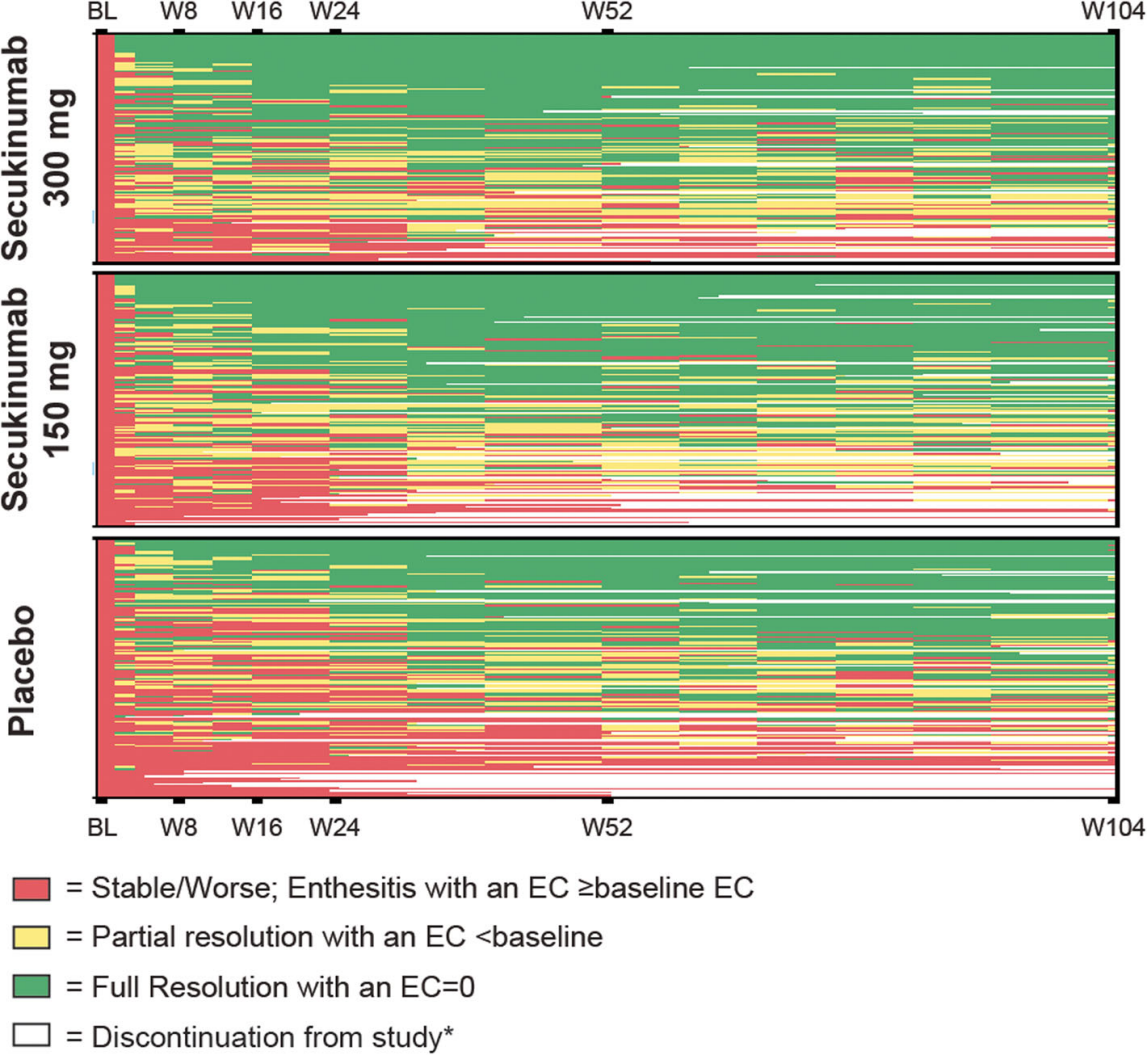
Heat map of enthesitis resolution by treatment group through week 104. The asterisk indicate discontinuation due to following reasons: adverse events, death, lack of efficacy, lost to follow-up, non-compliance with study treatment, physician decision, pregnancy, patient/guardian decision, and withdrawal of informed consent. Placebo patients switched therapies at week 16 or 24. All patients with enthesitis at baseline were followed until the end of study or discontinuation. BL, baseline, EC, enthesitis count; W, week

Heat map analysis showed that secukinumab-treated patients at individual levels had more resolution of EC than placebo patients at week 24, which was sustained through week 104 (Fig. 4).

### Development of enthesitis in patients without enthesitis at baseline

**Fig. 5:**
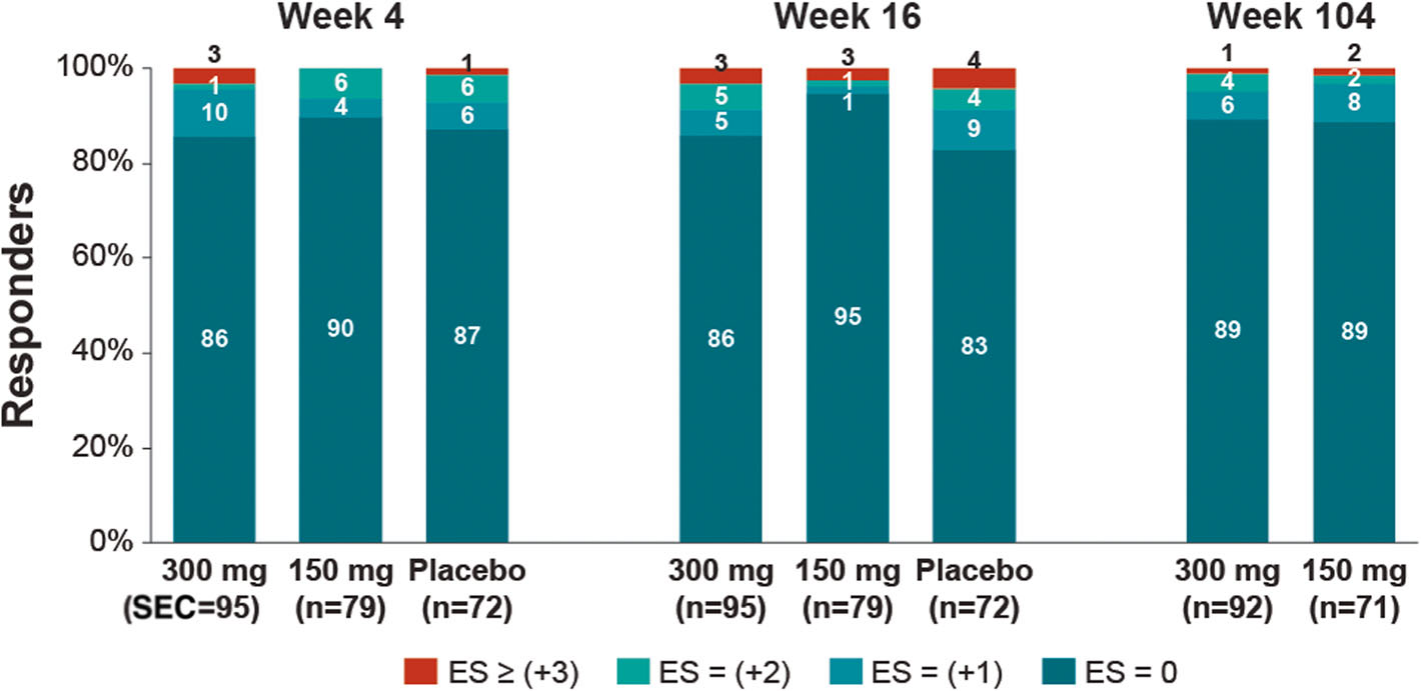
Change in enthesitis sites with secukinumab 300 mg and 150 mg over week 104 in patients with no enthesitis at baseline. Data presented are as observed. Number of evaluable patients at week 4—90 (300 mg), 78 (150 mg), and 70 (placebo); at week 16—92 (300 mg), 78 (150 mg), and 70 (placebo); and at week 104—83 (300 mg) and 62 (150 mg). ES, enthesitis site; *n*, number of patients with no enthesitis at weeks 4, 16, and 104

The majority of patients without enthesitis at baseline did not develop enthesitis at week 16 (86%, 95%, and 83% in the secukinumab 300 mg, 150 mg, and placebo groups, respectively), which was maintained at week 104 (89% with both secukinumab 300 mg and 150 mg) (Fig. 5).

### Relationship between enthesitis status and outcomes across multiple clinical domains

At week 16, patients with enthesitis at baseline treated with secukinumab 300 and 150 mg showed better outcomes compared with placebo for ACR20 (51.6% and 44.3% vs 19.5%, respectively), ACR50 (31.4% and 21.0% vs 7.5%), PASI 90 (66.5% and 51.8% vs 13.1%), DAS28-CRP (− 1.4 and − 1.0 vs − 0.5), HAQ-DI (− 0.5 and − 0.3 vs − 0.2), and SF-36 PCS (6.1 and 3.5 vs 2.3). Improvements were generally greater with secukinumab 300 mg than 150 mg for all efficacy outcomes (Table 2). In patients without enthesitis at baseline, secukinumab 300 and 150 mg again showed higher responses than placebo for ACR20 (48.8% and 60.4% vs 13.8%), ACR50 (32.6% and 32.9% vs 4.1%), and PASI 90 (56.4% and 48.9% vs 10.8%), with reduced disease activity (DAS28-CRP − 1.3 and − 1.5 vs − 0.4) and improved physical function and HRQoL (HAQ-DI − 0.4 and − 0.5 vs − 0.2; SF-36 PCS 6.1 and 7.0 vs 2.0) (Table 2). The improvements with secukinumab 300 and 150 mg were sustained or further improved over 104 weeks for most outcome measures in patients with and without enthesitis (Table 2). Unadjusted analysis showed similar trends for improved and sustained efficacy outcomes until week 104 in secukinumab-treated patients (Additional file 1: Table S1).

**Table 2:** Adjusted efficacy outcome measures in patients with or without enthesitis at baseline

Outcome measures	Week	With enthesitis at baseline			Without enthesitis at baseline		
		Secukinumab		Placebo (*N* = 163)	Secukinumab		Placebo (*N* = 72)
		300 mg (*N* = 144)	150 mg (*N* = 159)		300 mg (*N* = 95)	150 mg (*N* = 79)	
ACR20^a,b^	16	51.6	44.3	19.5	48.8	60.4	13.8
	104	55.2	59.1	–	57.6	59.8	–
ACR50^a,b^	16	31.4	21.0	7.5	32.6	32.9	4.1
	104	43.4	25.7	–	43.5	28.9	–
ACR70^a,b^	16	20.5	10.7	2.9	23.4	18.5	1.4
	104	26.7	17.0	–	32.8	18.9	–
PASI 90^a,c^	16	66.5	51.8	13.1	56.4	48.9	10.8
	104	46.2	34.9	–	52.5	24.8	–
PASI 75^a,c^	16	82.3	62.0	10.6	68.1	59.8	7.3
	104	88.5	87.4	–	95.5	80.1	–
HAQ-DI^d^	16	− 0.5	− 0.3	− 0.2	− 0.4	− 0.5	− 0.2
	104	− 0.4	− 0.3	–	− 0.5	− 0.5	–
SF-36 PCS^d^	16	6.1	3.5	2.3	6.1	7.0	2.0
	104	6.9	4.0	–	6.3	6.0	–
DAS28-CRP^d^	16	− 1.4	− 1.0	− 0.5	− 1.3	− 1.5	− 0.4
	104	− 1.7	− 1.5	–	− 1.9	− 1.7	–

Logistic regression (for binary variables) or ANCOVA (for continuous variables) were performed as a function of the patient characteristics at baseline (treatment group, enthesitis status, gender, DAS28-CRP, HAQ-DI, TNFi-status), and the interaction between treatment groups and enthesitis status

*ACR* American College of Rheumatology, *DAS28-CRP*; 28-Joint Disease Activity Score count using C-reactive protein, *HAQ-DI* Health Assessment Questionnaire Disability Index, *LS* least square, *n* number of evaluable patients, *N* total number of patients, *PASI* Psoriasis Area and Severity Index, *SF-36 PCS* Short Form 36 Physical Component Summary score

^a^Response, %

^b^At week 16/104, *n* = 144/132 (secukinumab 300 mg), 159/145 (secukinumab 150 mg), and 163 (placebo) with enthesitis and *n* = 95/91 (secukinumab 300 mg), 79/70 (secukinumab 150 mg), and 72 (placebo) without enthesitis at baseline

^c^At week 16/104, *n* = 66/56 (secukinumab 300 mg), 82/62 (secukinumab 150 mg), and 63 (placebo) with enthesitis and *n* = 38/34 (secukinumab 300 mg), 46/36 (secukinumab 150 mg), and 30 (placebo) without enthesitis at baseline (psoriasis subset)

^d^LS mean change from baseline

## Discussion

The current knowledge on the treatment of enthesitis is limited. Most randomized, controlled trials in PsA focus on polyarticular disease, which accounts only for a subgroup of PsA. To date, no randomized, controlled study has been specifically designed to evaluate the treatment of enthesitis. Notably, most clinical trials in which enthesitis indices were applied to assess enthesitis outcomes among patients displaying this symptom at baseline were not adequately powered to assess enthesitis [25–28].

The objectives of this pooled analysis from the FUTURE 2 and FUTURE 3 studies were to describe the demographics and clinical characteristics of the patients who had both polyarticular disease as per inclusion criteria and baseline enthesitis, and to assess the efficacy of secukinumab on resolution of enthesitis in patients with PsA over 2 years. The present analysis showed that the 65% of PsA patients who were diagnosed with enthesitis at baseline had more severe disease activity and reduced physical function compared with patients without enthesitis. This is consistent with data from the Corrona registry in PsA showing a higher disease activity, a poorer functional status, a worse quality of life, and greater patient-reported pain and fatigue in patients with enthesitis compared to those without enthesitis [13]. Enthesitis, therefore, is an important clinical domain of PsA that serves as an indicator of disease severity and high disease burden.

Clinically, enthesitis is invariably perceived as tenderness at entheses with entheseal swelling being uncommon. Different indices have been developed to help assess and measure enthesitis. The most commonly used indices for the assessment of enthesitis include the Spondyloarthritis Research Consortium of Canada (SPARCC), Maastricht ankylosing spondylitis enthesitis score (MASES), and LEI [24], which are clinically validated, reliable, and sensitive to change. The LEI and SPARCC are more specific for enthesitis in PsA, examining 6 and 16 sites of enthesitis, respectively. The LEI is easy to perform, and when compared with MASES, SPARCC, and Berlin indices, LEI correlates most consistently with the clinical parameters of disease activity in patients with PsA [24, 29]. Furthermore, LEI is capable of distinguishing between patients with and without active PsA [23]. In the present analysis, we used the clinical resolution of enthesitis (EC = 0) which is a very stringent outcome in contrast with the LEI change from baseline used in most PsA clinical trials with TNFi [27, 28, 30]. TNFi were shown to improve peripheral enthesitis as assessed by clinical indices of enthesitis in PsA [25–27, 30]. However, little is known on the median time to resolve enthesitis, and the magnitude of response by enthesitis severity, by time since diagnosis, or after switch to other TNF inhibitors [27, 28, 30] as well as development of enthesitis over time in patients with no enthesitis at baseline.

In the overall population, patients with enthesitis at baseline treated with secukinumab showed higher rates of full resolution of enthesitis compared with placebo at week 16, which further improved at week 104. The magnitude of response was higher in patients treated with secukinumab 300 than 150 mg. Similarly, secukinumab showed early and sustained resolution of enthesitis in TNFi-naïve and TNFi-IR patients through 2 years, with higher response in TNFi-naïve than TNFi-IR patients. Resolution of enthesitis data from this pooled analysis are in line with previously reported enthesitis data from the FUTURE 5 trial where an early and sustained resolution was observed up to 2 years (week 24, 61.4% [*P* < 0.0001] and 54.6% [*P* < 0.001] with secukinumab 300 and 150 mg, respectively, vs 34.4% with placebo; week 104, 78% and 80.3% with secukinumab 300 and 150 mg, respectively) [31, 32].

These post hoc analyses illustrate for the first time in the literature the kinetics of enthesitis response to a biologic treatment as assessed by time to resolution of enthesitis, complemented with a shift analysis from baseline to weeks 24, 52, or 104, and heat map analysis. Secukinumab provided faster resolution of enthesitis compared with placebo in the overall population, as well as in both TNFi-naïve and TNFi-IR patients, with faster response in TNFi-naïve than TNFi-IR patients. These data are encouraging because enthesitis is known to be very painful and limiting, and can last for prolonged periods if not treated appropriately. Although full resolution took longer time in the more refractory TNFi-IR patients, the median time for achieving complete resolution was within the first 3 months. Secukinumab 300 mg showed faster resolution compared with 150 mg in the overall population and TNFi-naïve patients.

The shift analysis of enthesitis from baseline to week 24 by degree of severity of EC at baseline showed higher rate of full resolution in patients with mild to moderate enthesitis (EC = 1 or 2) with secukinumab 150 and 300 mg than placebo at week 24, with increased resolution at weeks 52 and 104. In contrast, no difference in full resolution of enthesitis was observed at week 24 between placebo and secukinumab 150 or 300 mg in patients with EC of 3–6 and proportions of patients from this group who had full resolution at weeks 52 and 104 were lower than in patients with less severe enthesitis. This highlights that achievement of full resolution in patients with more severe disease takes longer time and is more difficult to achieve. Heat map analysis helped visualizing EC resolution at individual level and extends the findings of the shift analysis by visually showing that from week 24 to 104, most patients treated with secukinumab sustain resolution of enthesitis.

The majority of patients without baseline enthesitis (89%) did not develop enthesitis over 2 years of treatment with secukinumab 300 or 150 mg. This supports the hypothesis that IL-17A inhibition may prevent the development of new enthesitis sites as well as providing sustained resolution of enthesitis in patients with baseline enthesitis.

TNFi studies have indicated that PsA patients with enthesitis are a difficult-to-treat population with a lower odds of achieving minimal disease activity (MDA) compared to patients without enthesitis [33]. Indeed, the absence of enthesitis is a predictor of MDA in patients treated with TNFi [34]. In contrast, secukinumab-treated patients with PsA showed higher ACR and PASI responses, reduced disease activity, and improved physical function and HRQoL versus placebo, regardless of enthesitis status at baseline. A greater magnitude of improvement in signs and symptoms, physical function, and quality of life was observed in patients with enthesitis at baseline treated with secukinumab 300 mg than with 150 mg. Importantly, the response rates in patients with and without enthesitis at baseline indicate that secukinumab 300 mg showed similar efficacy on different composite endpoints in both populations. This is in line with a pooled efficacy analysis from FUTURE 2–5 using machine learning which showed a greater response with secukinumab 300 mg over 150 mg in patients with baseline enthesitis [35]. These improvements reported across all endpoints were sustained or further increased over 104 weeks of secukinumab treatment, which were consistent with the FUTURE 1 and 5 studies [17, 31, 32].

The GRAPPA recommendations issued in 2016 mentioned non-steroidal anti-inflammatory drugs and physiotherapy as initial treatment and TNFi as the first-line biologics for enthesitis in PsA patients [1]. Limited enthesitis data with secukinumab were available when the GRAPPA recommendations were issued. Data from TNFi studies are difficult to compare with the present pooled analysis as they used either LEI change from baseline or different scoring systems such as MASES and SPARCC [27, 36]. A head-to-head trial comparing secukinumab and adalimumab, which includes the assessment of enthesitis as a key secondary endpoint, will be reported in the future (NCT02745080). In another head-to-head trial of an IL-17 inhibitor (ixekizumab) versus adalimumab (SPIRIT-P1), ixekizumab showed higher resolution of enthesitis (as measured by LEI) than adalimumab at week 24 (ixekizumab 80 mg once every 4 weeks, 43%, and ixekizumab 80 mg once every 2 weeks, 39%, vs adalimumab, 33%) [37]. The recently published pooled data from the SPIRIT-P1 and SPIRIT-P2 studies with ixekizumab showed significant resolution of enthesitis at week 24 versus placebo (ixekizumab 80 mg once every 4 weeks, 39%, and ixekizumab 80 mg once every 2 weeks, 35%, vs placebo, 21%) [38]. Data from the FUTURE studies [19, 20, 32] along with this pooled analysis provide comprehensive evidence supporting the efficacy of secukinumab on enthesitis in PsA patients.

The potential limitations of the current analysis include its post hoc nature. Clinical enthesitis was not an inclusion criteria in the FUTURE studies. EC by use of the LEI (with only six sites) entails a probability of missing peripheral enthesitis at other sites. Scoring of enthesitis in the medial femoral condyle may be difficult because of joint swelling. Clinical indices such as LEI may not be specific, especially when there is overlap with fibromyalgia, mechanical injury, or tendinitis. Power Doppler Ultrasound was not performed at baseline to confirm the clinical assessment of enthesitis, and X-ray did not evaluate the impact on structural damage at baseline and up to 2 years. Another limitation is that the observed data analyses do not account for dropouts or missing data. There was no placebo group beyond weeks 16/24, and given the post hoc nature of this study, only numerical but no statistical comparisons were done between treatment groups. No additional analysis was done to assess the frequency of each enthesitis site at baseline and if any of them was more sensitive to treatment. The impact of secukinumab on resolution of enthesitis using imaging will be assessed in two multicenter, randomized, placebo-controlled trials which are currently ongoing: (1) the ULTIMATE study (NCT02662985) will use ultrasound to demonstrate the time course of response to secukinumab of enthesitis in biologic naïve PsA patients; (2) the ACHILLES study (NCT02771210) will use magnetic resonance imaging to evaluate the efficacy of secukinumab on resolving Achilles tendon enthesitis in patients with active PsA and axial spondyloarthritis despite current or previous NSAID, DMARD, or TNFi exposure.

## Conclusion

This post hoc analysis further extends the evidence for an early and sustained efficacy of secukinumab on enthesitis in patients with PsA irrespective of previous TNFi exposure. Secukinumab 300 mg was associated with greater efficacy on enthesitis than 150 mg notably in patients with higher baseline EC. Although patients with enthesitis at baseline had higher disease activity, secukinumab improved outcomes across multiple clinical domains of PsA as early as week 16 and maintained efficacy over 2 years, with responses being similar to patients without enthesitis, especially with the secukinumab 300 mg dose.

## Supplementary information

**Additional file 1: Table S1.** Unadjusted efficacy outcome measures in patients with or without enthesitis at baseline.

## Data Availability

The datasets generated and/or analyzed during the current study are not publicly available. Novartis is committed to sharing with qualified external researchers access to patient-level data and supporting clinical documents from eligible studies. These requests are reviewed and approved based on scientific merit. All data provided are anonymized to respect the privacy of patients who have participated in the trial in line with applicable laws and regulations. The data may be requested from the corresponding author of the manuscript.

## Abbreviations

ACR: American college of rheumatology
ANCOVA: Analysis of covariance
CASPAR: ClASsification criteria for Psoriatic ARthritis
DIP: Distal interphalangeal joint
DMARDs: Disease-modifying anti-rheumatic drugs
DAS28-CRP: 28-Joint Disease Activity Score using C-reactive protein
EC: Enthesitis count
ES: Enthesitis sites
FR: Full resolution
GRAPPA: Group for Research and Assessment of Psoriasis and Psoriatic Arthritis
HAQ-DI: Health Assessment Questionnaire Disability Index
HRQoL: Health-related quality of life
IL: Interleukin
LEI: Leeds Enthesitis Index
LOCF: Last observation carried forward
MASES: Maastricht ankylosing spondylitis enthesitis score
MDA: Minimal disease activity
NSAIDs: Non-steroidal anti-inflammatory drugs
PASI: Psoriasis area and severity index
PsA: Psoriatic arthritis
PR: Partial resolution
s.c: Subcutaneous
SF-36 PCS: Short Form 36 Physical Component Summary score
SPARCC: Spondyloarthritis Research Consortium of Canada
TNFi: Tumor necrosis factor inhibitor
