# Supplementary material for: Secukinumab efficacy on resolution of enthesitis in psoriatic arthritis: pooled analysis of two phase 3 studies
Source: Arthritis Res Ther. 2019 Dec 4;21:266. doi: 10.1186/s13075-019-2055-z (PMC6894120; doi:10.1186/s13075-019-2055-z)
Supplement: Supplementary file 1 — Additional file 1: Table S1. Unadjusted efficacy outcome measures in patients with or without enthesitis at baseline. [file 13075_2019_2055_MOESM1_ESM.docx]

**Table S1. Unadjusted efficacy outcome measures in patients with or without enthesitis at baseline**

|  | | **With enthesitis at baseline** | | | **Without enthesitis at baseline** | | |
| --- | --- | --- | --- | --- | --- | --- | --- |
| **Outcome Measures** | **Week** | **SEC 300 mg**  **(N=144)** | **SEC 150 mg**  **(N=159)** | **PBO**  **(N=163)** | **SEC 300 mg**  **(N=95)** | **SEC 150 mg**  **(N=79)** | **PBO**  **(N=72)** |
| **ACR20^a,b^** | **16** | 53.5 | 46.5 | 19.6 | 53.7 | 64.6 | 18.1 |
|  | **104** | 56.8 | 52.4 | – | 62.6 | 62.9 | – |
| **ACR50^a,b^** | **16** | 31.3 | 21.4 | 6.7 | 35.8 | 35.4 | 5.6 |
|  | **104** | 44.7 | 24.8 | – | 47.3 | 34.3 | – |
| **ACR70^a,b^** | **16** | 16.0 | 8.2 | 1.8 | 21.1 | 16.5 | 1.4 |
|  | **104** | 26.5 | 15.2 | – | 34.1 | 21.4 | – |
| **PASI 90^a,c^** | **16** | 50.0 | 36.6 | 7.9 | 42.1 | 37.0 | 6.7 |
|  | **104** | 67.9 | 59.7 | – | 73.5 | 44.4 | – |
| **PASI 75^a,c^** | **16** | 78.8 | 59.8 | 11.1 | 65.8 | 56.5 | 6.7 |
|  | **104** | 76.8 | 77.4 | – | 91.2 | 66.7 | – |
| **HAQ-DI^d^** | **16** | −0.5 | −0.3 | −0.2 | −0.5 | −0.5 | −0.2 |
|  | **104** | −0.5 | −0.4 | – | −0.5 | −0.6 | – |
| **SF-36 PCS^d^** | **16** | 6.4 | 3.7 | 2.5 | 6.5 | 7.4 | 2.6 |
|  | **104** | 7.4 | 4.3 | – | 6.6 | 6.9 | – |
| **DAS28-CRP^d^** | **16** | −1.5 | −1.0 | −0.5 | −1.4 | −1.6 | −0.5 |
|  | **104** | −1.7 | −1.6 | – | −2.0 | −1.9 | – |
| ^a^Response, %  ^b^At Week 16/104, n=144/132 (secukinumab 300 mg), 159/145 (secukinumab 150 mg) and 163 (placebo) with enthesitis and n=95/91 (secukinumab 300 mg), 79/70 (secukinumab 150 mg) and 72 (placebo) without enthesitis at baseline  ^c^At Week 16/104, n=66/56 (secukinumab 300 mg), 82/62 (secukinumab 150 mg) and 63 (placebo) with enthesitis and n=38/34 (secukinumab 300 mg), 46/36 (secukinumab 150 mg) and 30 (placebo) without enthesitis at baseline (psoriasis subset)  ^d^LS mean change from baseline  ACR, American College of Rheumatology; DAS28-CRP; Disease activity score 28-joint count using C-reactive protein; HAQ-DI, Health assessment questionnaire disability index; LS, least square; n, number of evaluable patients; N, total number of patients; PASI, Psoriasis Area and Severity Index; PBO, placebo; SEC, secukinumab; SF-36 PCS, Short Form 36 Physical Component Summary score | | | | | | | |
